# Pituitary–Adrenal Responses and Glucocorticoid Receptor Expression in Critically Ill Patients with COVID-19

**DOI:** 10.3390/ijms222111473

**Published:** 2021-10-25

**Authors:** Dimitra A. Vassiliadi, Alice G. Vassiliou, Ioannis Ilias, Stylianos Tsagarakis, Anastasia Kotanidou, Ioanna Dimopoulou

**Affiliations:** 1 Department of Endocrinology, Diabetes and Metabolism, National Expertise Centre for Rare Endocrine Diseases, Evangelismos Hospital, 106 76 Athens, Greece; dimitra.vas@googlemail.com (D.A.V.); stsagara@otenet.gr (S.T.); 2 First Department of Critical Care Medicine & Pulmonary Services, School of Medicine, National & Kapodistrian University of Athens, Evangelismos Hospital, 106 76 Athens, Greece; alvass75@gmail.com (A.G.V.); akotanid@med.uoa.gr (A.K.); 3 Department of Endocrinology, Helena Venizelos Hospital, 115 21 Athens, Greece; iiliasmd@yahoo.com

**Keywords:** HPA, GCR, COVID-19, critical illness

## Abstract

The hypothalamus–pituitary–adrenal (HPA) axis was described as the principal component of the stress response 85 years ago, along with the acute-phase reaction, and the defense response at the tissue level. The orchestration of these processes is essential since systemic inflammation is a double-edged sword; whereas inflammation that is timely and of appropriate magnitude is beneficial, exuberant systemic inflammation incites tissue damage with potentially devastating consequences. Apart from its beneficial cardiovascular and metabolic effects, cortisol exerts a significant immunoregulatory role, a major attribute being that it restrains the excessive inflammatory reaction, thereby preventing unwanted tissue damage. In this review, we will discuss the role of the HPA axis in the normal stress response and in critical illness, especially in critically ill patients with coronavirus disease 2019 (COVID-19). Finally, a chapter will be dedicated to the findings from clinical studies in critical illness and COVID-19 on the expression of the mediator of glucocorticoid actions, the glucocorticoid receptor (GCR).

## 1. Introduction

The activation of the hypothalamus–pituitary–adrenal (HPA) axis is a principal component of the stress response evoked by systemic inflammation [1], and occurs in conjunction with the acute-phase reaction and the tissue level defense process. The orchestration of these processes is essential since systemic inflammation is a double-edged sword; whereas inflammation that is timely and appropriate in magnitude is beneficial [2], exuberant systemic inflammation incites tissue damage with potentially devastating consequences [3]. Apart from its beneficial cardiovascular and metabolic effects, excess cortisol originating by HPA axis activation exerts significant immunoregulatory effects [4], with a major attribute being that it restrains the excessive inflammatory reaction, thereby preventing unwanted tissue damage.

Severe acute respiratory syndrome coronavirus 2 (SARS-CoV-2) is the cause of coronavirus disease 2019 (COVID-19), a disease with a wide range of clinical manifestations; although most infected subjects remain asymptomatic or with a mild disease, a number develop a respiratory illness of variable severity that, in some cases, can progress to acute respiratory distress syndrome (ARDS), multi-organ failure, and death. The pathophysiology of ARDS and multi-organ injury is considered to involve the development of an inflammatory organ insult based on accumulating evidence that a subgroup of patients displays an aggressive inflammatory response, with markedly elevated inflammatory markers and pro-inflammatory cytokines, a condition termed “cytokine storm” [5]. Given cortisol’s ability to restrain excessive inflammatory responses, a relevant question is whether the HPA axis activation in COVID-19 patients is appropriate for such an inflammatory challenge. 

## 2. The Normal Stress Response

### 2.1. The Stress System

The hypothalamus and pituitary gland form a unit that controls the function of the thyroid, gonads, and adrenals. Inner hypothalamic neurons serve neuroendocrine functions (most hypothalamic cells participate in other functions, such as temperature regulation, sleep, food intake, sexual behavior, and emotional state). The pituitary gland lies at the skull base (in the sella turcica); it is divided into the anterior (adenohypophysis) and posterior lobe (neurohypophysis). The posterior lobe of the pituitary gland is of neural origin, while the anterior pituitary gland originates in Rathke’s pouch; furthermore, cells from Rathke’s pouch form an intermediate pituitary lobe that disperses in the anterior lobe and produces propiomelanocortin (POMC) and adrenocorticotrophin (ACTH) [6]. A local vascular network (portal circulation) directly connects the hypothalamus with the anterior pituitary gland [7]. Reverse flow from the pituitary to the hypothalamus may enable local feedback/neuroendocrine control [8]. The adrenal glands are located at the upper pole of the kidneys; their size varies. The adrenal glands consist of an outer layer, the cortex, and an internal, the medulla. In adults, the cortex makes up 90% of the adrenal gland and surrounds the centrally located medulla. Sympathetic preganglionic fibers (whose trunks rest on the lower thoracic and upper lumbar spine) and parasympathetic fibers from the abdominal branch of the pneumogastric nerve form networks that penetrate the adrenal cortex passing into the medulla. This design can play a role in regulating adrenal cortical function during stressful situations, providing a neural pathway that connects the adrenal glands to the hypothalamus [6].

### 2.2. Normal Function of the HPA Axis

**Figure 1:**
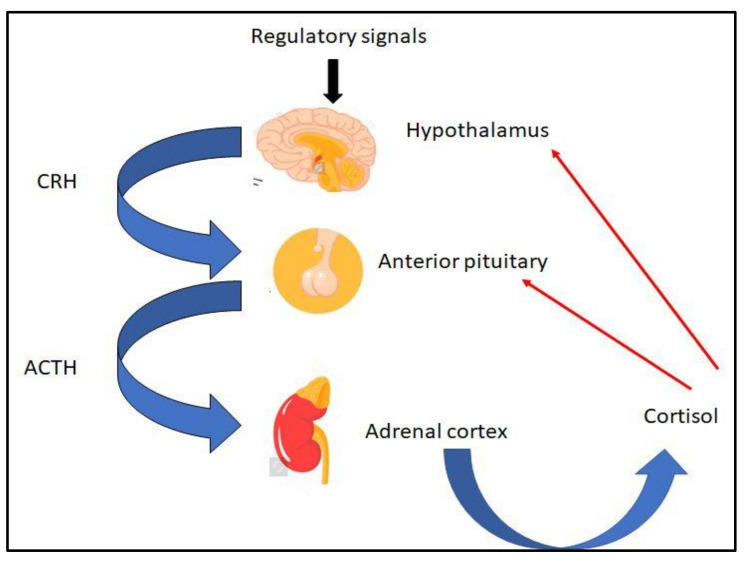
The normal stress response. Cortisol production in the adrenals is regulated by the secretion of ACTH from the pituitary gland, which depends primarily on pulses of CRH, secreted by the hypothalamus. Cortisol then inhibits CRH and ACTH secretion. ACTH: adrenocorticotrophin; CRH: corticotropin-releasing hormone.

In healthy individuals, cortisol production in the adrenals is regulated by the secretion of ACTH from the pituitary gland, which depends primarily on pulses of corticotropin-releasing hormone (CRH) secreted by the hypothalamus. Neuropeptides, cytokines, and vasoactive peptides appear to be involved in regulating corticosteroid secretion regardless of ACTH [9,10]. CRH is a polypeptide consisting of 41 amino acids and is derived from a 196-amino acid precursor molecule. It has a half-life of approximately 60 min. Upon its secretion, it activates the release of ACTH and other products of the POMC precursor molecule from the anterior pituitary gland. CRH-producing neurons have been identified in the paraventricular and paraoptic nuclei of the hypothalamus, while they have also been found in the placenta. The rate and magnitude of CRH secretion are regulated by the central nervous system, as well as plasma cortisol and ACTH levels (negative feedback regulation) [6]. Antidiuretic hormone and angiotensin II augment the effect of CRH on the pituitary secretion of ACTH, whereas oxytocin inhibits it. ACTH is a 39-amino acid-long peptide hormone derived from a larger precursor molecule, POMC. Apart from CRH, normal ACTH secretion occurs with food intake; its increase marks the end of nighttime sleep. Arginine vasopressin (AVP), which is secreted by the hypothalamus, also stimulates ACTH production, but always in the presence of CRH. Stress activates the CRH–ACTH–cortisol system and can eliminate its daily secretory rhythm. The biological half-life of ACTH in the circulation is lower than 10 min. The action of ACTH on the adrenal cortex leads to the fast synthesis and secretion of steroid hormones; within a few minutes the levels of circulating hormones increase. Chronic stimulation with ACTH leads to hyperplasia and hypertrophy of the adrenal glands. Figure 1 depicts the normal stress response.

Circadian rhythms are an integral part of the adaptive mechanisms of humans to their environment. The HPA follows a circadian rhythm [11]. Plasma concentrations of ACTH and cortisol tend to be high in the morning and low in the evening. Maximum ACTH levels are usually present between 4:00 and 6:00 in the morning, while peak cortisol levels occur around 6:00 to 8:00 in the morning. Cortisol peaks in the morning until noon with a range between 9–12 μg/dL, while, at 16:00–20:00 in the afternoon, the levels drop approximately to 50% of the morning values and continue to decline. Both ACTH and cortisol are released in a pulsatile fashion every 30–120 min. The pulses occur regardless of whether the basal cortisol concentration is high or low, resulting in a wide range of cortisol fluctuations [12]. Apart from the rhythmic secretion of CRH from the hypothalamus, food intake, exposure to light or darkness, cerebral energy needs, hepatic/renal function or impairment, substance abuse, and physical or emotional stressors influence the HPA’s circadian rhythm.

Cortisol inhibits CRH and ACTH secretion. This negative feedback effect can be rapid, intermediate, and prolonged [8,13]. Rapid negative feedback is sensitive to the rate of change in cortisol concentration regardless of its levels. This phase is rapid, and ACTH levels decrease within minutes after the increase in cortisol; it lasts less than ten minutes (which points to a non-genomic mechanism). Apparently, intermediate negative feedback occurs also via a non-genomic mechanism. Prolonged negative feedback depends on the absolute levels of cortisol; it occurs via genomic mechanisms (classical glucocorticoid receptors) [14]. In addition to cortisol (long feedback), ACTH inhibits CRH secretion from the hypothalamus (short loop feedback).

Total plasma cortisol includes the free fraction (<5%) and the protein-bound fraction, mainly cortisol-binding globulin (transcortin, CBG), and, to a lesser extent, albumin [15]. The free cortisol fraction is the biologically active one. An increase in CBG is observed in the presence of high estrogen levels (e.g., during pregnancy or on oral contraceptive pills). In the kidneys, cortisol is converted to inactive cortisone. The liver is also important in the metabolism of cortisol; it is mainly in this organ that cortisone is converted to cortisol. 

### 2.3. Pathophysiology of the Stress System

The systems involved in the adaptation/reaction to conditions that threaten homeostasis are the HPA, the noradrenergic alert system, and the sympathetic nervous system [16]. Cognitive assessment of a situation as being stressful, organic signals into the blood stream (hormones, nutrients, inflammatory molecules), and information from peripheral nerves are combined by the brain to produce a series of specific and scalable actions in the context of the stress response [17]. The most easily recognizable response is the immediate activation of the sympathetic nervous system. This triggers the release of noradrenaline from the locus coeruleus in the brain stem and sympathetic ganglia and adrenaline from the adrenal medulla [6]. The subsequent neuroendocrine response to stress is the activation of the HPA axis, leading to an increase in the secretion of cortisol [18]. CRH-secreting neurons and central nervous system noradrenergic neurons interact, mainly through α1-noradrenergic receptors and CRH receptors, respectively. In addition, various factors, such as serotonin and acetylcholine, stimulate CRH- and noradrenergic-neurons, while both are subject to negative feedback from glucocorticoids as well as from other substances (gamma amino butyric acid-GABA, ACTH, or opioids). There is also the interaction of the adrenal medulla with the cortex. Stress exposure, through the action of cytokines (interleukin-1-IL-1, IL-6, tumor necrosis factor α-TNFα), activates the HPA, while catecholamines act as releasing factors for cytokines from immune cells via β-adrenergic receptors [10].

## 3. Pituitary–Adrenal Responses in Critical Illness

Critical illness refers to life-threatening conditions requiring invasive monitoring and high levels of vital organ support, including mechanical ventilation within an intensive care unit (ICU). Such examples are severe infections (i.e., sepsis or septic shock), trauma, extended burns, and post-major surgery. Hospital mortality for patients admitted to the ICU has decreased significantly over the past decades despite an increase in the severity of illness. This is due to a better understanding of the underlying complex pathophysiology of critical illness and technological and therapeutic advances [19]. 

Several adaptive mechanisms are triggered to support the vital functions and to recover homeostasis during critical illness. Among these, an appropriate activation of the HPA axis and cortisol response are crucial for survival. Both high and low cortisol levels have been associated with increased mortality; high levels eventually represent severe stress, whereas low levels are due to a blunted cortisol response [20]. The elevated plasma cortisol concentrations are driven by increased cortisol production. Activation of the HPA axis results in increased secretion of both CRH and AVP from the hypothalamic paraventricular nucleus. CRH and AVP act synergistically to stimulate the release of ACTH from the anterior pituitary. Cytokines, as well as catecholamines, angiotensin II, serotonin, and vasoactive intestinal peptide (VIP), also act directly on the pituitary to stimulate ACTH secretion. CRH and ACTH mediate cortisol release in the acute phase, whereas non-ACTH-mediated pathways are involved during prolonged critical illness. In turn, ACTH stimulates the production of cortisol, adrenal androgens, and, to a lesser extent, mineralocorticoid from the adrenal cortex. Other mechanisms implicated in maintaining high cortisol levels during critical illness are the reduction of the negative feedback that cortisol exerts on the hypothalamus–pituitary. Moreover, stress results in decreased secretion of CBG, leading to an increase in free cortisol concentrations. Moreover, the normal circadian rhythm of cortisol secretion, characterized by the highest concentrations in the morning, is lost during critical illness. Finally, high cortisol availability is also driven by a reduction of cortisol metabolism due to the impaired activation of the enzymes responsible, and by the prolongation of the half-life of circulating cortisol in the presence of renal dysfunction. The increase in cortisol levels results in multiple effects (metabolic, cardiovascular, and anti-inflammatory) aiming to maintain homeostasis during critical illness [21,22]. 

In 2008, a task force proposed the term critical illness-related corticosteroid insufficiency (CIRCI) to describe HPA axis dysfunction during critical illness. CIRCI is characterized by an inadequate production of cortisol in relation to increased demands during periods of severe stress and is accompanied by an exaggerated and protracted pro-inflammatory response [23]. CIRCI occurs as a result of a decrease in adrenal steroid production or tissue resistance to glucocorticoids due to impaired glucocorticoid receptor alpha (GCR-α) activity. The reported prevalence rates of CIRCI in critically ill patients vary according to the characteristics of the study population and the diagnostic criteria used. CIRCI may occur in any critical state, i.e., head trauma, burn, major surgery; however, it seems that CIRCI is more prevalent in sepsis and septic shock. Rates as high as 60–90% have been documented in patients with septic shock. Patients with CIRCI do not have specific clinical or laboratory findings. It usually presents as hypotension refractory to fluids and vasopressors in patients with septic shock or as unexplained hypotension. There are no established diagnostic criteria for CIRCI. Adrenal insufficiency in critically ill patients is best defined as a delta total serum cortisol of <9 µg/dL after ACTH administration (250 µg) or a random total cortisol of <10 µg/dL. A more reliable tool would be the measurement of serum free cortisol; nevertheless, it is not readily available in clinical practice, and cut-off values have not been established for critically ill patients. Salivary free cortisol has a good correlation with serum free cortisol; however, obtaining a sample can be challenging in critically ill patients. No other dynamic tests, such as insulin hypoglycemia, the metyrapone, or CRH tests, are approved in severe illness. The mechanisms leading to the dysfunction of the HPA axis during critical illness are complex and poorly understood. A subset of patients may have structural damage to the adrenal gland from either hemorrhage or infarction, which may result in long-term adrenal dysfunction. Moreover, a number of drugs may cause CIRCI; the most common mechanisms relate to the increased metabolism of cortisol (dilantin, phenobarbital, and rifampin), interference with steroidogenic enzymes (ketoconazole and etomidate), or agents that inhibit central regulation (e.g., opioids). However, many critically ill patients develop reversible failure of the HPA axis without any structural damage to the adrenal gland, the pituitary, or the hypothalamus [9]. The benefit of treatment with glucocorticoids at this time seems to be limited to patients with vasopressor-dependent septic shock. Hydrocortisone in a dose of 200 mg/day in four equal doses, or a continuous infusion in a dose of 240 mg/day (10 mg/h) for about 7 days is recommended in septic shock. Tapering the steroids once vasopressors are no longer needed is proposed. Studies have shown a beneficial effect of steroids in such patients in terms of the ability to discontinue vasopressors and possibly wean patients from mechanical ventilation, whereas survival does not seem to improve. Fludrocortisone is optional in CIRCI, while dexamethasone is not recommended. The role of low-dose hydrocortisone in patients with sepsis and other clinical situations in the ICU remains to be determined [24].

## 4. Pituitary–Adrenal Responses in COVID-19 Patients

There are several mechanisms that may result in potentially inadequate cortisol production in COVID-19 patients. Dysregulation of the HPA axis in those more severely affected may be encountered as part of the development of CIRCI, as has been described in other conditions of critical illness [9]. There are, however, several additional theoretical mechanisms by which COVID-19 patients may be at an increased risk of adrenal insufficiency, and more prone to the development of CIRCI when in critical condition. Two important viral receptors through which SARS-CoV-2 enters host cells are angiotensin-converting enzyme 2 (ACE2) and transmembrane protease serine 2 (TMPRSS2). Thus, tissues expressing these proteins are more likely to be affected. In fact, the presence of both ACE2 and TMPRSS2 has been documented in many endocrine tissues, including the adrenal zonae fasciculata and reticularis [25,26], as well as the hypothalamus and pituitary [27], making them possible direct targets of SARS-CoV-2. SARS-CoV-2 has been shown to infect multiple extrapulmonary organs in hamsters, including the adrenal gland. More specifically, the adrenal gland presented severe inflammation throughout the organ and also exhibited adrenal medulla atrophy [28]. COVID-19 is associated with an increased rate of pulmonary or extrapulmonary thrombotic complications [29], and adrenal injury may also occur through bilateral adrenal non-hemorrhagic or hemorrhagic infarction. Although incidental computed tomography (CT) findings compatible with adrenal infarction have been reported in 23% of patients with severe COVID-19 [30], the vast majority with bilateral involvement, clinically relevant cases are sparse with only nine reported so far [31]. Of note, two of these cases had positive antiphospholipid antibodies prior to COVID-19 diagnosis, suggesting a link between the presence of antiphospholipid syndrome and the risk of bilateral adrenal infarction [31].

Autopsies in patients who died after being infected by SARS-CoV, a virus closely related to SARS-CoV-2, have shown degeneration and necrosis of adrenocortical cells, whereas the presence of SARS-CoV was documented in the adrenal glands [32], implying a direct cytopathic effect. However, in a small study of deceased COVID-19 patients, examination of the adrenals showed mainly an acute fibrinoid necrosis of small vessels, mostly affecting arterioles in the adrenal parenchyma, capsule, and periadrenal adipose tissue [33]. Subendothelial vacuolization and apoptotic debris without significant signs of inflammation, parenchymal infarctions, or thrombosis were additional findings. In another autoptic study of fatal COVID-19 cases, the authors identified SARS-CoV-2 and its replication in the adrenal glands, which co-localized with ACE2 and TMPRSS2, mainly in epithelial but also in mesenchymal and endothelial cells [26]. No specific (histo-)morphologic alterations in the adrenals could be assigned to the SARS-CoV-2 infection; they mainly observed chronic inflammation with perivascular distribution. It should be noted that autopsy studies involve a very limited number of patients so far, and are hampered by the fact that they only include deceased patients, thus referring to those more severely affected with multi-organ failure.

Adrenal insufficiency may also occur due to hypothalamic–pituitary insult. SARS-CoV2 affects the brain, causing encephalitis, respiratory difficulties, and anosmia [34,35], possibly through olfactory and ACE2-related pathways [36]. A recent study in male mice established that the S1 subunit of the spike protein of SARS-CoV-2 crosses the blood–brain barrier and is taken up by brain regions that include the cortex, hypothalamus, and hippocampus, areas of particular importance for HPA axis control [37]. In small autoptic studies in patients infected with SARS-CoV, the somatotroph, thyrotroph, and corticotroph pituitary cells’ number, as well as the respective hormone immunoreactivity, were reduced; the opposite was observed for mammotroph and gonadotroph pituitary cells [38]. Whether there are similar alterations with SARS-CoV-2 infection remains unknown. Of note, in a postmortem study of COVID-19 patients, areas of necrosis/infarction were seen in one out of the nineteen pituitaries examined [39]. Besides direct organ injury, an interesting, albeit largely speculative, theory was initially proposed for SARS-CoV, but it may equally involve SARS-CoV-2 given their high degree of analogy [40]. It implicates the presence of homology between specific amino acid sequences of the viruses and the ACTH molecule. Production of antibodies against these specific virus proteins may theoretically interfere with endogenous ACTH, hindering its action on the adrenals [27]. However, currently, there is no evidence to support this intriguing hypothesis.

Beyond hypotheses, however, there are limited clinical data on adrenal function in COVID-19 patients. Clinical studies reporting on the adrenal function in SARS-CoV-2 affected subjects should be approached keeping in mind that COVID-19 is a highly heterogenous disease, with regard to severity, underlying comorbidities, and administered medications, especially glucocorticoids, etomidate, or azole antifungals, all of which may have a significant impact on cortisol levels. In the first and largest study, Tan et al. measured cortisol levels in 403 non-critically ill COVID-19 patients within 48 h of hospital admission; their levels were significantly higher than in patients admitted to the hospital for other reasons, indicating a marked and appropriate acute cortisol stress response [41]. In fact, the higher the cortisol levels, the greater was the hazard of mortality, in consistence with numerous previous studies in non-COVID-19 patients, suggesting that cortisol is a marker of disease severity. In critically ill patients, the results were similar; in a study of 144 COVID-19 critically ill patients, high levels of cortisol were observed in the patients with more severe illness and reduced survival [42]. A positive association with disease severity was also confirmed in the study by Das et al., who reported higher cortisol levels in COVID-19 patients with moderate to severe disease compared to those with mild disease [43]. At the same time, cortisol levels were not elevated in asymptomatic and mild cases. A small study reported cortisol levels below 300 nmol/L (10 μg/dL) in a significant number of asymptomatic/mild cases, a finding that was not observed in repeated testing after a few days in most patients [44]. 

ACTH levels were measured in the study by Das et al. [43]. They tended to be lower, but without reaching statistical significance, in those with moderate to severe disease, consistent with the published evidence of dissociation between cortisol and ACTH levels in severe illness [45]. This ACTH and cortisol dissociation is attributed to the dependence of cortisol secretion from other than ACTH factors, such as cytokines, which are expected to be higher in the more severe cases. Reduced peripheral cortisol metabolism, resulting in increased systemic half-life, represents an additional ACTH-independent mechanism of increased cortisol levels with a consequent feedback reduction of ACTH [46]. 

Overall, the existing evidence does not support an association of COVID-19 with the presence of reduced adrenal function. If anything, high levels of cortisol have been consistently observed corresponding to the severity of COVID-19, with the higher levels predicting an adverse outcome. Therefore, glucocorticoid replacement therapy is not routinely recommended. Stress doses are, however, imperative for patients with known or clinically possible adrenal insufficiency (i.e., those on chronic steroid use), or those with high clinical suspicion, ideally after appropriate testing, according to the recent European Society of Endocrinology (ESE) guidance for adrenal insufficiency and COVID-19 [47]. 

A distinction should be made between the administration of glucocorticoids for stress coverage in patients with adrenal insufficiency and the administration of pharmacological dosages aiming to exploit its potent anti-inflammatory properties. Although in the beginning of the COVID-19 pandemic the pharmacological use of steroids was avoided based on previous negative experience from other sever respiratory infections, such as SARS [48] and Middle East Respiratory Syndrome (MERS) [49], it soon became evident that it may offer a survival benefit, especially when administered at the stage where immunopathological elements prevail over vigorous viral replication. Therefore, the appropriate choice of time, dose, and patient is mandatory. Currently, 6 mg of dexamethasone administered intravenously once daily for up to 10 days is recommended based on the results of the RECOVERY trial [50], which supports its use in appropriately selected patients, that is, those patients with COVID-19 who are receiving respiratory support.

## 5. Glucocorticoid Receptor Expression in Critically Ill Non-COVID-19 and COVID-19 Patients

### 5.1. The Glucocorticoid Receptor and Glucocorticoid Resistance

**Figure 2:**
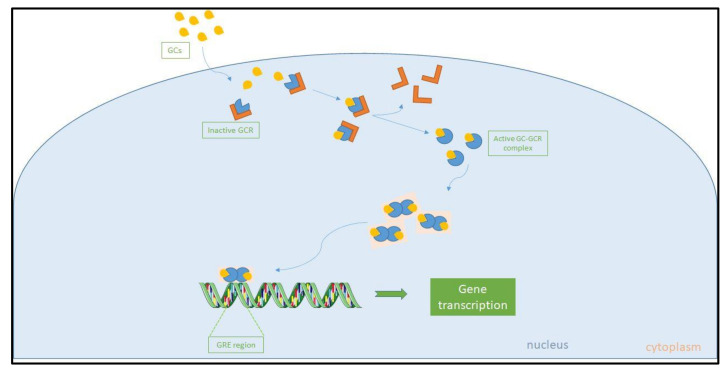
Cortisol signaling through the glucocorticoid receptor. Cortisol signaling is mediated by the glucocorticoid receptor. The receptor–cortisol complex translocates from the cytosol to the nucleus, where it exerts transcriptional activation or repression, the end result being the inhibition of the inflammatory response. GC: Glucocorticoid; GCR: Glucocorticoid receptor; GC–GCR: Cortisol–glucocorticoid receptor complex; GRE: Glucocorticoid responsive element.

The immunological, metabolic, and hemodynamic actions of glucocorticoids (GCs) are mediated by a ubiquitous intracellular receptor, the glucocorticoid receptor (GCR). Alternative splicing of the primary GCR transcript gives rise to two highly homologous isoforms [51]. GCR-α is the functionally active receptor that binds to cortisol. After binding, the GCR–cortisol complex translocates from the cytosol to the nucleus, where the complex exerts transcriptional activation or repression by directly binding to genes that contain GC responsive elements [52]. The end result is the inhibition of the pro-inflammatory response [53,54]. On the contrary, GCR-β has not been well-explored. It is well-known to suppress GCR-α activity and is unable to bind natural or synthetic ligands [55,56,57]. Figure 2 diagrammatically represents cortisol signaling via GCR. 

In 2016, the current Sepsis-3 guidelines suggested the administration of hydrocortisone in septic shock patients who are resistant to fluid resuscitation and vasoactive agents [58]. Not all patients seemed to respond to this treatment, suggesting the existence of resistance to glucocorticoids. GC resistance is defined as the inability of GCs to exert their effects on target tissues [59]. It is characterized by decreased sensitivity of immune cells to GCs, which, under normal conditions, terminate the inflammatory response [60]. Therefore, apart from the cortisol levels, the effect of cortisol on tissues must also be taken into account. How tissues respond to cortisol, reflecting the extent of cortisol’s effect, might depend on the levels of GCR expression, the subtype expressed, or the affinity of the receptor for cortisol in a specific target cell [61]. Such an example is the increased expression of GCR-β mRNA in certain tissues in inflammatory diseases; the increased GCR-β mRNA expression has been linked with decreased GC sensitivity [62].

### 5.2. GCR Expression and Glucocorticoid Resistance in Critically Ill Patients

Glucocorticoid resistance may be a consequence of decreased GCR mRNA and protein expression, reduced GCR affinity for the ligand and nuclear translocation, and/or DNA binding. Most of the data on GC resistance in critical illness originates from experimental septic models. It seems that the overall mRNA expression of GCR-α is down-regulated, while mRNA expression of GCR-β is up-regulated in sepsis [63,64,65,66,67,68,69,70,71].

In clinical studies involving humans, mostly cortisol availability has been studied in critical illness; only a few studies have explored the role of GCR in septic adults, yielding conflicting results. The data on GCR mRNA expression and critical illness in humans point towards GC resistance, especially in sepsis. GC resistance has been described in a cohort of septic patients, who exhibited reduced GCR-α and elevated GCR-β mRNA expression levels compared to healthy subjects; these results prompted the authors to suggest that treatment with steroids might aggravate GC resistance in patients with increased GCR-β mRNA levels [72]. In a different study, a transient increase in GCR-β mRNA expression was seen in sepsis, and, moreover, serum from the septic patients could induce GC resistance in vitro [73]. Reduced GCR-α mRNA expression [74] and protein levels [75] have also been reported in sepsis. Decreased GCR-α and increased GCR-β receptor numbers have been detected in heart and liver biopsies from septic patients [63]. In septic shock, GCR protein expression increased, while GCR binding capacity decreased, proposing that it is the reduced GCR binding capacity and not the number of receptors that interferes with the response to exogenous or endogenous GCs [76]. On the other hand, one study showed that GCR number and affinity did not differ in septic patients compared to control subjects, suggesting that GCs could be effective in the hemodynamic compensatory phase of sepsis [77]. Increased GCR-α mRNA expression has been shown in the acute phase of sepsis, questioning the need for exogenous steroids at this phase [78]. Only one study has demonstrated decreased cytosolic GCR protein levels, and subsequent down-regulation of cortisol binding, in ventilated critically ill patients [79]. Finally, our group showed that polymorphonuclear cells, isolated from steroid-free critically ill patients, exhibited a highly variable expression of both GCR isoforms [80]. GCR expression and HPA axis function showed a biphasic response during critical illness, causing an imbalance; this dissociation of reduced GCR expression and elevated cortisol prompted us to suggest that critical illness is characterized by an abnormal stress response [81]. In pediatric critically ill cohorts, decreased α:β GCR mRNA expression ratio and increased GCR-β mRNA expression was shown to be related to illness severity in infants with bronchiolitis [82]. Another study showed that the total and cytoplasmic, but not nuclear, GCR protein levels were significantly lower in critically ill children compared to healthy controls [83]. Critically ill children with cardiovascular dysfunction and increased illness severity had lower GCR-α protein expression in CD4 and CD8 lymphocytes [84]. In another pediatric septic shock cohort, decreased expression of the GCR-α protein correlated with poor outcomes from septic shock, particularly in those patients with high serum cortisol [85].

### 5.3. GCR Expression in COVID-19

Data on COVID-19 and GCR are limited. Our group was able to demonstrate that critically ill COVID-19 patients exhibit increased mRNA expression of both GCR-α and the GC-inducible gene, glucocorticoid-inducible leucine zipper (GILZ), as well as increased serum cortisol levels, compared to critically ill patients without COVID-19 but comparable disease severity. The up-regulation of GILZ mRNA correlated with the up-regulation of GCR-α mRNA, but not with serum cortisol levels, IL-6, or IL-10 [86]. Our results are indicative of the strong activation of endogenous cortisol response to SARS-CoV-2 that might, however, be insufficient to prevent death. In a very recent study, single-cell RNA sequencing data, from the bronchoalveolar lavage fluid of severe COVID-19 patients receiving corticosteroid therapy, revealed the co-expression of GCR-α and IL-6 mRNAs; moreover, GCR-α mRNA expression was found decreased in severe COVID-19 patients compared to mild patients. The authors suggested that this may reflect a pathologic down-regulation of this endogenous immunomodulatory mechanism that could be restored pharmacologically with corticosteroid therapy [87].

## 6. Conclusions

A functioning HPA axis resulting in appropriate cortisol secretion is essential for surviving acute illness. This is of particular relevance to COVID-19 on account of cortisol’s beneficial ability to counteract the excessive inflammatory reaction, a cardinal pathophysiologic mechanism of organ injury in severe COVID-19. Data until now suggest that the adrenal response is appropriate in the majority of patients, and corresponds to the severity of stress. With regard to cellular cortisol signaling, there is no evidence of resistance to cortisol action since patients with COVID-19 exhibit increased expression of both GCR-α and GILZ compared to non-COVID-19 patients with critical diseases of comparable severity. It is, however, possible that the attained level of GILZ expression is still inefficient to achieve maximum immunosuppression, providing grounds for corticotherapy in patients with COVID-19. In fact, the significant survival benefit of glucocorticoids in selected COVID-19 patients observed in the RECOVERY clinical trial [50] is mainly attributed to the potent immunosuppressive effects of the pharmacological administration of glucocorticoids.

## Data Availability

Not applicable.
